# *Dictyostelium* Cells Migrate Similarly on Surfaces of Varying Chemical Composition

**DOI:** 10.1371/journal.pone.0087981

**Published:** 2014-02-06

**Authors:** Colin P. McCann, Erin C. Rericha, Chenlu Wang, Wolfgang Losert, Carole A. Parent

**Affiliations:** 1 Department of Physics, University of Maryland College Park, College Park, Maryland, United States of America; 2 Laboratory of Cellular and Molecular Biology, Center for Cancer Research, NCI, NIH, Bethesda, Maryland, United States of America; 3 Institute for Physical Science and Technology, University of Maryland, College Park, Maryland, United States of America; 4 Biophysics Graduate Program, University of Maryland, College Park, Maryland, United States of America; King's College London, United Kingdom

## Abstract

During cell migration, cell-substrate binding is required for pseudopod anchoring to move the cell forward, yet the interactions with the substrate must be sufficiently weak to allow parts of the cell to de-adhere in a controlled manner during typical protrusion/retraction cycles. Mammalian cells actively control cell-substrate binding and respond to extracellular conditions with localized integrin-containing focal adhesions mediating mechanotransduction. We asked whether mechanotransduction also occurs during non-integrin mediated migration by examining the motion of the social amoeba *Dictyostelium discoideum*, which is thought to bind non-specifically to surfaces. We discovered that *Dictyostelium* cells are able to regulate forces generated by the actomyosin cortex to maintain optimal cell-surface contact area and adhesion on surfaces of various chemical composition and that individual cells migrate with similar speed and contact area on the different surfaces. In contrast, during collective migration, as observed in wound healing and metastasis, the balance between surface forces and protrusive forces is altered. We found that *Dictyostelium* collective migration dynamics are strongly affected when cells are plated on different surfaces. These results suggest that the presence of cell-cell contacts, which appear as *Dictyostelium* cells enter development, alter the mechanism cells use to migrate on surfaces of varying composition.

## Introduction

The ability of cells to migrate on surfaces of differing composition is crucial during many biological and pathological responses, such as immune responses, wound healing and cancer metastasis [1]. However, the extent by which cells adhere to a given substrate varies widely, depending on the cell type. In general, eukaryotic cells use two distinct types of migration, each of which are distinguished by the nature and the extent of cell-substrate adhesion [2]. Mesenchymal cells, such as fibroblasts, exhibit strong cell-substrate adhesion and form characteristic focal adhesions during migration. In contrast, amoeboid cells, such as neutrophils and dendritic cells, have very weak cell-substrate adhesions and do not form large focal adhesions during migration. Integrins represent the major transmembrane receptor by which mammalian cells sense their environment and adhere to surfaces [3]. Cell-substrate adhesion, much like cell migration, is regulated through changes in cytoskeletal forces, which are mainly mediated through the polymerization of actin into filaments and the assembly of myosin II [4]. While integrins do not directly interact with actin, a group of adapter proteins are known to mediate the signals from integrins to the actin cytoskeleton. Talin is an adapter protein that binds to both integrins and actin [3].

The social amoebae *Dictyostelium discoideum* is exposed to a variety of surfaces as the cells enter a developmental program and transition from single cell to collective cell migration [5]. During growth, these amoebae migrate on a substrate to track down and phagocytose bacteria. When starved, they enter a differentiation program that allows the cells to survive harsh environmental conditions. They do so by secreting and chemotaxing toward adenosine 3′, 5′ cyclic monophosphate (cAMP) signals, causing a head-to-tail migration pattern resulting in aggregates that later differentiate into a multicellular organism. The molecular components that control cell-substrate adhesion in *Dictyostelium* during both growth and development remain largely unknown. A handful of adhesion receptors have been identified in this organism [6], [7], and although two of them, SibA and SibC, have homologies with mammalian integrin β chains (i.e. an extracellular Von Willebrandt A domain, a glycine-rich transmembrane domain and a highly conserved cytosolic domain that interacts with talin [8], [9]), no *bona fide* integrin homologue is expressed [10]. Yet, *Dictyostelium* cells express two homologues of talin: talin A and talin B, which have distinct functions. Talin B harbors a unique C-terminal domain homologous to the villin headpiece and is required for multicellular morphogenesis [11], while talin A is more related to mammalian talin [12] and is required during single cell migration for cell-particle as well as cell-substrate interactions [13].

In the present study, we set out to determine the migratory ability of chemotactic competent *Dictyostelium* cells when plated on surfaces of varying chemical composition. We studied the adhesion and movement of both individual and groups of cells on four surfaces that exhibit different hydrophobicity and charge and assessed the role of actin, myosin II and talin on these parameters. Our study is therefore aimed at assessing the role of cell-surface contact and the underlying cytoskeleton during chemotaxis and collective cell migration.

## Materials and Methods

### Cell Culture

WT *Dictyostelium* (strain AX3 and AX2), adenylyl cyclase A null cells (*aca* ^−^) [14], myosin-II null cells (*myoII* ^−^) [15] and talin A null cells (*talin A* ^−^) [12] were grown in HL-5 medium to 4–5×10^6^ cells/ml. For all experiments, cells were developed for 4.5 (WT) or 5 (*aca* ^−^, *myoII* ^−^, *talin A* ^−^) hrs in development buffer (DB; 5 mM Na_2_HPO_4_, 5 mM NaH_2_PO_4_, pH 6.2, 2 mM MgSO_4_ and 0.2 mM CaCl_2_) at 2×10^7^ cells/ml, with exogenous pulses of 75 nM cAMP every 6 min, as previously described [16]. Unless otherwise noted, cells were taken from development or growth and centrifuged at 500 g for 3 min. The supernatant was aspirated and the pellet was washed twice with phosphate buffer (PB; 5 mM Na_2_HPO_4_, 5 mM NaH_2_PO_4_, pH 6.2) and finally resuspended in PB and processed according to the assay performed. Experiments requiring Latrunculin A treatment involved resuspending cells in PB with 5 µM Latrunculin A (Invitrogen) for 5 min before placing them in a chamber in which the buffer contained 5 µM Latrunculin A. For the Latrunculin A titration experiments, cells were resuspended in PB with different concentration of Latrunculin A (0 (only PB), 5 µM, 1.5 µM, 0.5 µM, 0.25 µM) for 5 min before placing them in a chamber where the buffer contained the same concentration of Latrunculin A.

### Surface Preparation

For most experiments Lab-Tek eight-chamber slides (Lab-Tek, Nunc) were used. Each chamber was washed with 400 µl of 1 M HCl for 15 min and then rinsed 3 times with water. For BSA (Sigma) or PLL (Sigma) coatings, each chamber was incubated with 400 µl of 1% w/v solutions for 4 hrs. FCC (tridecafluoro-1,1,2,2-tetra hydrooctyl dimethyl chlorosilane; Gelest SIT8170.0) was allowed to vapor deposit on a dry chamber for 4 hrs in a vacuum chamber. Coverslips coated with high-density polyethylene glycol (PEG) were purchased from MicroSurfaces, Inc. (Austin, TX; www.proteinslides.com). After coating, surfaces were washed 3 times with water before use. Static contact angles were measured with 5 µl drops of water with a goniometer.

### Shaking Assay

The shaking adhesion assay was performed as follows [16], [17]. 300 µl containing 5×10^5^ cells were allowed to settle for 15 min in an 8-well Lab-Tek chamber (1 cm^2^ surface area) pre-treated with a given surface coating. The buffer was replaced to eliminate non-adherent cells. The cells were then placed on an orbital shaker with a 1 cm radius of gyration at 200 rpm. After 15 min, the buffer was aspirated, and the number of cells in the supernatant was counted using a hemocytometer. Finally, buffer was added to the chamber and repeatedly aspirated, and the number of cells in this buffer was counted. The surfaces were visually inspected to ensure that no cells remained after the final aspiration.

### Microscopy

F-actin labeling experiments were carried out with the following protocol. Cells were allowed to migrate for 30 min, fixed in 2% formaldehyde and 0.2% glutaradehyde for 10 min, and rinsed twice. Next, 2 µM TRITC-phalloidin in 0.2% Triton was added to each well for 30 min, and the samples were placed under aluminum foil. Finally the samples were rinsed 5 times in PBS [18].

Population aggregation assays were performed on a Zeiss Axiovert S100 microscope equipped with a CoolSnap HQ CCD camera (Roper Scientific) and an automated moveable stage using a 2.5× (NA 0.075) objective. Phase-contrast imaging was adjusted so that objects (cells and streams) appeared bright on a black background, which provided sufficient contrast for automated tracking routines to easily identify objects. Images were acquired every min, and all time-lapse imaging lasted for at least 90 min.

Interference reflection microscopy (IRM) was performed on a Zeiss Meta 510 microscope with a 40× (NA 1.3) objective. A HeNe laser provided the excitation line of 488 nm, which was then imaged in two channels: transmitted light and <510 nm for IRM signature. Images were acquired every 4 secs. Cell shapes, locations, and center-of-mass speeds were calculated from time-lapse images as described in the Image Analysis section. Polarization of a cell was calculated as: Polarization = (Perimeter)^2^/(4π Area ), giving a value of 1 for a circular object and a value greater than 1 for any other shape. To determine the contact area per cell for cells in groups, individual cells forming or entering a group were manually tracked so that the number of cells in the group was known; then the contact area of the group was measured.

### Image Analysis

Images were binarized using ImageJ software (National Institutes of Health; http://rsbweb.nih.gov/ij/). For low-magnification phase-contrast images, the background was subtracted using a rolling-ball algorithm and the remaining image thresholded. For high-magnification BF images, a variance filter was applied, followed by thresholding and binary erosion. For IRM images, a bandpass filter was used prior to thresholding to smooth out unevenness in illumination. Identification of cells in phase-contrast images, as well as tracking, was carried out using custom Matlab (The Mathworks, Natick, MA) code. This allowed fully automated cell tracking as the software kept track of individual moving cells and only counted those cells that were not part of a larger group. No subjective measures were used to include or exclude specific cells from the population analyses (see below). Contact ratio was calculated by dividing the IRM area by the BF area. Cell centroid statistics were calculated as described in [16].

Quantifying aggregate shape and size was performed as follows: all individuals and groups of cells in the image were identified, and the morphological skeleton of the resultant shapes was computed. In brief, this process removes as much of an object as possible while still retaining its characteristic shape. For example, a branching structure will become a one-pixel wide branching structure, while a circle will become a single pixel. This differentiates between objects of the same number of pixels but different spatial extents (see Fig. S4A in File S1 for an illustration). Once this skeletonization was performed, the average size of the skeletons of objects larger than one cell were computed, as a function of time. This showed whether or not the largest objects were branching streams (large branching spatial extent, large skeleton) or aggregates (small round spatial extent, with a much smaller skeleton). This quantification of aggregation and streaming provided a robust metric to determine the aggregation pattern. We then compared the maximum spatial extent of the skeletons from all four surfaces.

## Results and Discussion

Cell-surface adhesion in *Dictyostelium* is thought to be mediated through nonspecific membrane interactions [19], [20], [21], and hence altering surface chemistry, such as varying hydrophobicity and charge, is expected to play a significant role in modulating cell-surface adhesion and migration [22]. We therefore examined *Dictyostelium* adhesion and migration on five distinct surfaces: acid-washed glass (GLASS), glass coated with bovine serum albumin (BSA), glass coated with poly-L-lysine (PLL), glass coated with a perfluorinated carbon chain (FCC), and glass coated with polyethylene glycol (PEG) (see Table S1 in File S1 and Material and Methods). On the PEG surface the cells neither adhered nor migrated (data not shown), and no further experiments were carried out on that surface.

### Nonspecific Adhesion is Actively Regulated

**Figure 1:**
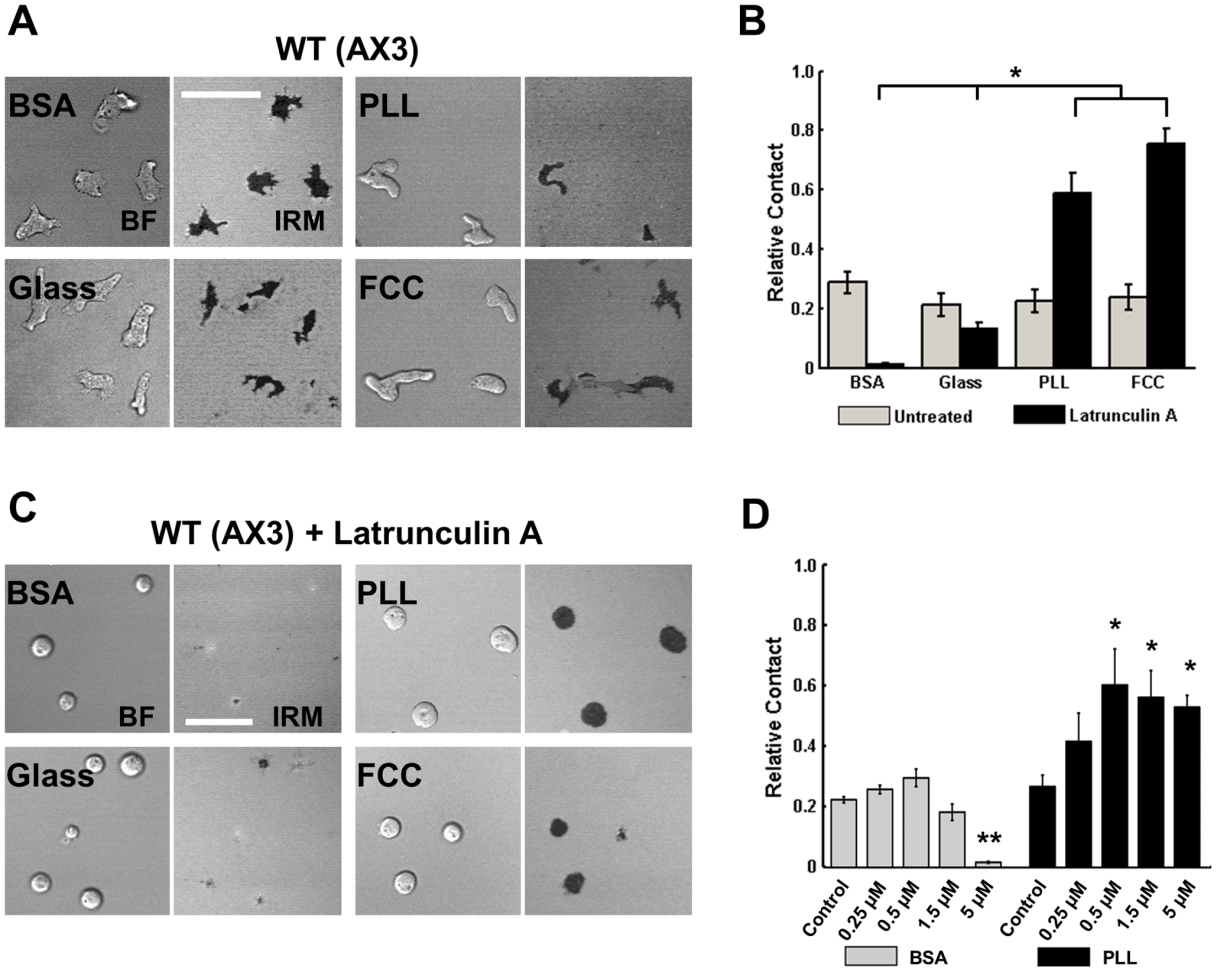
Cell-surface adhesion is actin-dependent. **A.** Representative bright field (BF; left half of image) and IRM (right half of image) images of WT (AX3) cells on the 4 different surfaces. Scale bar = 35 µm. **B.** Quantification of average percent of cell in contact on each surface, for WT (AX3) cells and WT cells treated with 5 µM Latrunculin A. **C.** Representative BF and IRM images of WT (AX3) cells treated with Latrunculin A and plated on the 4 different surfaces. Scale bar = 35 µm. **D.** Quantification of average percent of cell in contact on BSA and PLL surfaces. WT cells were treated with different Latrunculin A concentrations, as indicated. For B and D, error bars indicate SEM of three independent experiments, each with over 30 individual cells analyzed. For B, *indicates statistical significance (p<0.05; ANOVA, Tukey test). For D, *indicates statistical significance compared to the control condition (*: p<0.05, T-test; **: p<0.005; T-test).

We used Interference Reflection Microscopy (IRM) [23] to compare the relative contact area (the ratio of the steady-state contact area to projected bright-field area) for each of the different surface preparations. Surprisingly, we found that the surface composition did not change cell-surface contact area of wild type (WT) chemotaxis-competent cells (see Supplemental Material and Methods; Figs. 1A&B and S2A&B in File S1). As another means to study cell contact, we subjected the cells to a shaking adhesion assay and found similar results (Fig. S1A in File S1). These results are consistent with work from Loomis and colleagues, who carefully measured cell detachment under very controlled fluid shear flow and found comparable cell-surface adhesion strength on a wide range of surfaces [21]. The authors further observed that non-specific cell-substrate adhesion can be explained based on van der Waals attractions between cell surface glycoproteins and the substratum, and thus adhesion appears unrelated to chemical signaling pathways. Indeed we found that the various surfaces do not alter the adhesion dynamics of vegetative cells or cells that cannot spontaneously aggregate (*aca* ^−^ cells), although *aca* ^−^ cells were consistently more adherent compared to WT cells (Fig. S1A&B in File S1).

Once adhered on a surface, the cell-surface contact area results from a balance of cell-surface adhesion forces and forces generated by the actomyosin cortex [24], [25], [26], [27]. To compare cell-surface adhesion strengths on the different surfaces in the absence of the actomyosin cortex we visualized the contact area of Latrunculin A-treated cells. Latrunculin A-treated cells adopt a spherical shape and do not elongate or form protrusions [28]. We found a strong relationship between surface compositions and contact area in Latrunculin A-treated cells using both IRM measurements and the shaking adhesion assay (Figs. 1B&C and S1A in File S1). Cells plated on BSA-coated glass showed low contact area compared to uncoated glass, consistent with reports of low cell-surface adhesion on BSA [29]. Cells on PLL- or FCC-coated glass displayed higher contact areas, indicating higher cell-surface adhesion forces. These results establish that in the absence of actin assembly, when cells do not actively exert forces on surfaces, the different surfaces display a clear hierarchy of inherent adhesivities. Furthermore, we found that *Dictyostelium* cells are able to regulate forces generated by the actomyosin cortex to maintain optimal cell-surface contact area and adhesion on various surfaces.

To further examine the effect of actin assembly on cell-surface adhesion, we measured the relative contact area of cells treated with different concentrations of Latrunculin A. BSA and PLL coated surfaces were chosen as representative surfaces that respectively display lower and higher contact area after Latrunculin A treatment. Representative images showing phalloidin staining of Latrunculin A-treated cells on both BSA- and PLL-coated surfaces are shown in Fig. S3 in File S1. Interestingly, we found that cells plated on BSA were more resistant to actin disassembly compared with cells plated on PLL. As low as 0.5 µM Latrunculin A significantly increased the relative contact area of cells plated on PLL (Fig. 1D). In contrast, cells plated on BSA required a minimum of 5 µM Latrunculin A before a significant decrease in relative contact area could be measured (Fig. 1D). Indeed, we noticed that at equivalent Latrunculin A concentrations the phalloidin staining is weaker in cells plated on PLL, compared to BSA, particularly at 0.5 µM Latrunculin A (Fig. S3 in File S1). These findings indicate that (i) *Dictyostelium* cells regulate their surface contact area and cell-surface adhesion in an actin-dependent manner and that (ii) actin contributes differently on different surfaces to the balance between the force generated by surface adhesion and the force generated through the actomyosin cortex.

### Cells Migrate with Similar Speeds and Polarity When Plated on Chemically Distinct Surfaces

**Figure 2:**
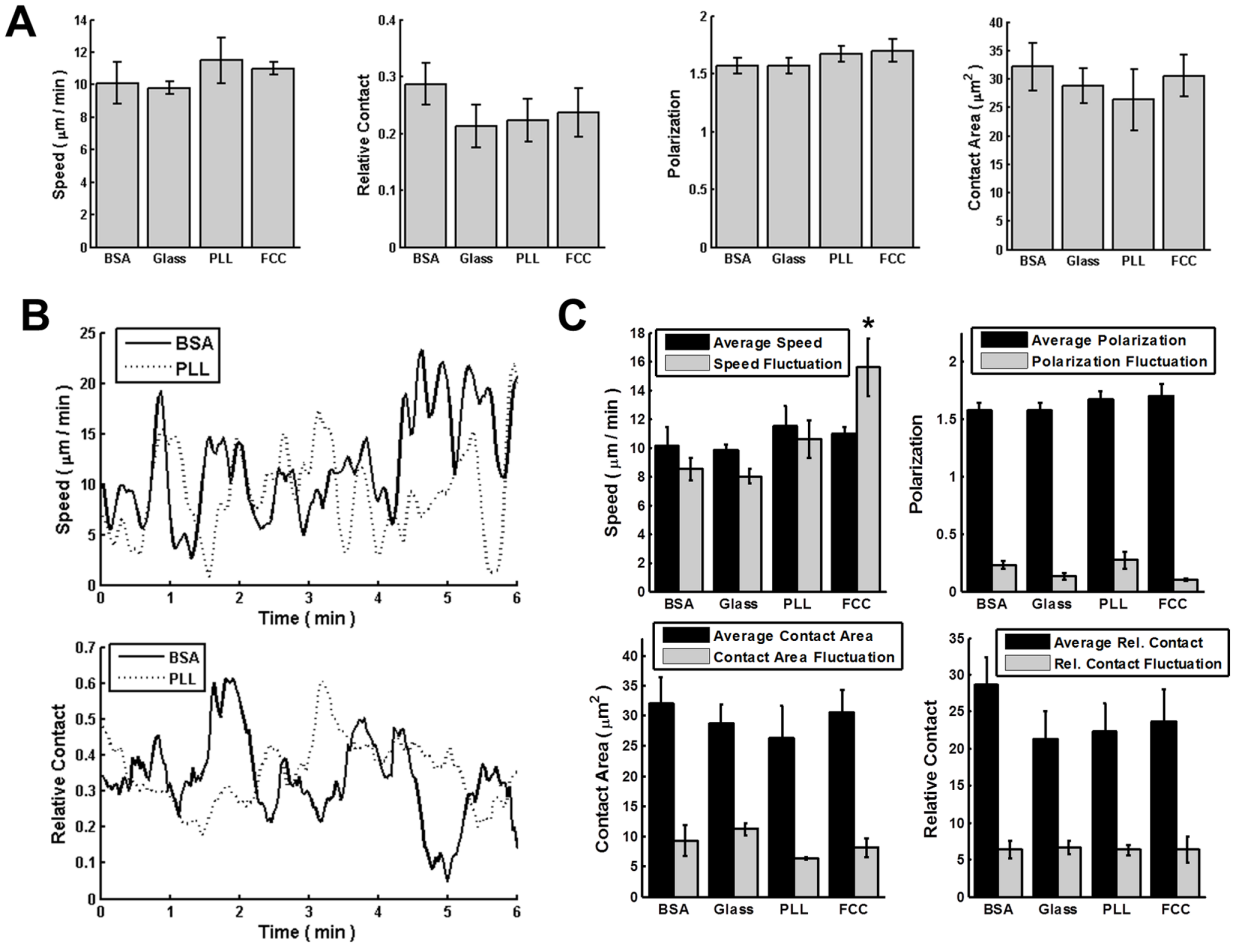
Cells migrate similarly on various substrates. **A.** Quantification of average speed, relative contact area, absolute contact area, and polarization (see Material and Methods) for WT (AX3) cells on each surface. Time-lapse images were collected for 90 min and each cell was tracked for at least 5 min. Error bars indicate SD of three independent experiments, each with over 30 individual cells analyzed. All values were not statistically different (p>0.05; ANOVA). **B.** Quantification of temporal speed and relative contact area for WT (AX3) cells during migration on BSA and PLL coated surface. Speed and relative contact are averaged over all cells at corresponding time point. **C.** Comparison of average speed, relative contact area, absolute contact area, and polarization with the fluctuation of all these four measurements. *indicates statistical significance on speed fluctuation compared to the other three surfaces (p<0.05; ANOVA, Tukey test).

Since cell migration is inherently more complex than cell-surface attachment, we hypothesized that while varying surface composition does not affect cell-surface adhesion or contact area in WT cells, it would alter migration speed or shape as previously observed for epithelial cells and keratocytes [30], [31]. Chemotactic-competent WT cells were plated on each of the four surfaces, allowed to spontaneously migrate, and their migration speed, shape, polarization, and contact area were quantified (see Material and Methods). As cells are known to deposit materials when they migrate [32], [33], potentially altering the surfaces, we only investigated cell migration for the first 30 min after plating. Remarkably, we measured no impact of surface composition on migration speed or polarization: cells migrated with an average speed of 10 µm/min on all surfaces examined and showed similar polarization and contact area (Fig. 2A). By plotting spontaneous measurements we observed large fluctuations in both speed and relative contact area as a function of time (Fig. 2B). We therefore asked whether the different surfaces could give rise to different fluctuations, while showing similar averages. Among all four surfaces tested we did not find strong differences in fluctuations of speed, cell polarization or surface contact, though the fluctuations in speed were measurably larger when cells were migrating on FCC (Fig. 2C). Our findings are consistent with simulations that focused on the role of cell-surface adhesion in *Dictyostelium* motility. The simulations showed that cell speed is roughly independent of cell-surface adhesive forces [19]. The model assumed that cells adhere to the surface via adhesive bridges and that changing the surface adhesion properties changes the rate of dissociation of the bridges, which does not represent a rate-limiting step for cell migration.

### Myosin-II is Required for Strong Membrane-surface Adhesions

**Figure 3:**
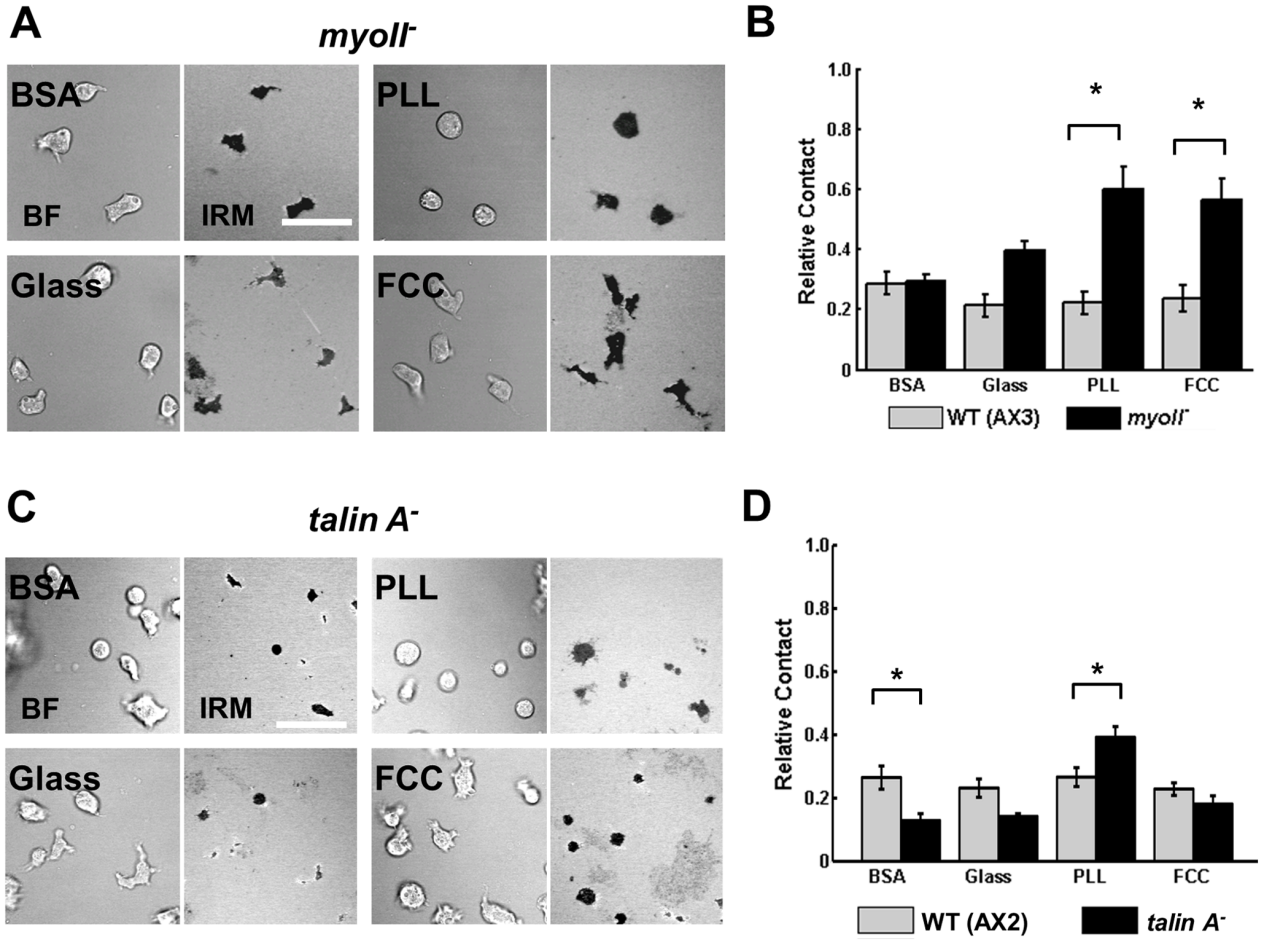
Myosin II and Talin A have different functions in cell-substrate adhesion. **A.** Representative BF (left panels) and IRM (right panels) images of *myoII* ^−^ cells when plated on various surfaces. Scale bar = 35 µm. **B.** Quantification of the contact area of WT (AX3) and *myoII* ^−^ cells. **C.** Representative BF (left panels) and IRM (right panels) images of *talin A* ^−^ cells when plated on various surfaces. Scale bar = 35 µm. **D.** Quantification of the contact area of WT (AX2) and *talin A* ^−^ cells. For B and D, Error bars indicate SEM of 3 independent experiments, each with over 30 individual cells analyzed. *indicates statistical significance (p<0.05; T-test).

The significant differences in cell-surface contact area in Latrunculin A-treated cells suggest that the actin cytoskeleton is involved in regulating membrane-surface adhesion. The actin cytoskeleton is known to be able to adapt to outside forces, mainly by controlling tension via molecular motors (myosin II) and by generating protrusions via actin polymerization [34]. We therefore examined how surface composition affects contact area in cells lacking myosin II (*myoII* ^−^ cells; [15]). Cells lacking myosin II have back retraction defects during migration [35] but, unlike Latrunculin A-treated cells, their actin cortex is able to maintain an elongated shape [34]. We found significant differences among the surface contact regions between *myoII* ^−^ and WT cells on two of the surfaces (Fig. 3A&B). On PLL and FCC, *myoII* ^−^ cells show an increase in cell-surface contact area compared to WT cells, indicating that the ability to contract is critical in maintaining consistent contact area on surfaces of higher adhesivity. A similar trend was also observed when cells are plated on glass. In contrast, on BSA cell-surface contact areas were similar for WT and *myoII* ^−^ cells, showing that contractility is not as critical on surfaces of lower adhesivity. Our findings suggest that cell-surface adhesion to less adherent surfaces (e.g. BSA) is regulated by cortical actin, and that this adhesion is independent of contractile ability, as *myoII* ^−^ cells spread similarly to WT cells on BSA, compared to Latrunculin A-treated cells on BSA (Fig. 1B&D).

### Talin A is Required for Weak Membrane-surface Adhesions

To further understand the molecular mechanisms that regulate cell adhesion and migration in *Dictyostelium*, we examined how surface composition affects contact area in cells lacking talin A (*talin A* ^−^ cells; [12]). As previously observed [13], we found that *talin A* ^−^ cells exhibit less cell-surface contact when plated on BSA compared to WT cells (Fig. 3C&D). In contrast, we could not measure significant differences in contact area between *talin A* ^−^ cells and WT cells when cells were plated on the other more adherent surfaces, except for PLL where the *talin A* ^−^ cells showed higher contact area (Fig. 3C&D). For these experiments, the *talin A* ^−^ parental cell line AX2 was used as a control. These cells behave similarly to AX3 cells on the various surfaces tested (Fig. S2A&B in File S1). Our findings reveal that talin A is required when cell-surface adhesion is enhanced by cortical actin (e.g. on BSA; see Fig. 1D).

### Cell-surface Adhesion Alters Collective Cell Migration

When cells migrate collectively they not only interact with their substrate, but also with each other. To test the competition between cell-cell and cell-surface adhesion, and to assess the effect of surface properties on collective cell motion, we imaged the early aggregation of starving *Dictyostelium* cells. During aggregation, cAMP signal relay results in the directed migration of cells in a head-to-tail fashion – a process known as streaming [36], [37]. Streams extending over the surface can be several cells wide and many cells in a stream are simultaneously in contact with other cells and the surface. This provides an ideal system to investigate the interplay between cell-cell and cell-surface adhesion.

**Figure 4:**
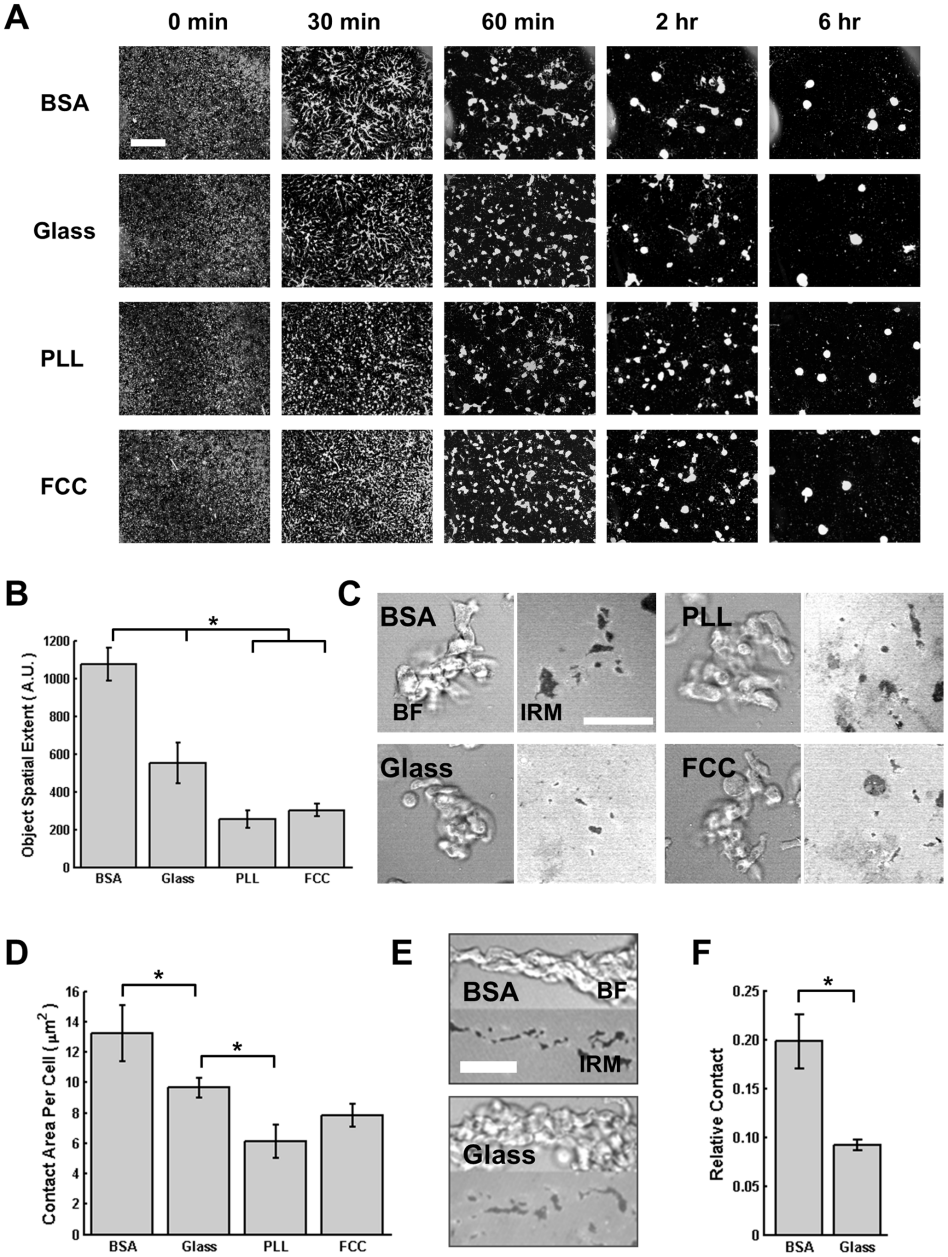
Surface Composition Affects Collective Cell Behavior. A. Montage of images depicting WT (AX3) cells aggregating on the 4 different surfaces, shown at 5 different times. Cells are white on a black background. Scale bar = 400 µm. B. Spatial extent of objects in time-lapse images. Error bars indicate SEM of 3 independent experiments (see Fig. S4 for quantification details). * indicates statistical significance (p<0.05; ANOVA, Tukey test). C. Representative BF (left panel) and IRM (right panel) images for groups of cells on each surface. Scale bar = 50 µm. D. Graph showing the quantification of the average cell-surface contact for each cell, for 3 groups on each surface. For each group, the number of cells in the group was counted and the total contact area was calculated, giving the average contact area for each cell. Error bars indicate SEM. *indicates statistical significance (p<0.05; ANOVA, Tukey test). E. Representative BF (top panels) and IRM (bottom panels) images of streaming cells on BSA or glass. Scale bar = 35 µm. F. Graph depicting the IRM contact area relative to the bright-field contact area, for time-lapse images of streams on BSA and glass. Error bars indicate SEM of 4 streams, all from independent experiments. *indicates statistical significance (p<0.05; T-test).

Cells were plated on the different surfaces and aggregate formation was recorded for several hours (Movie S1). Initially, the cells are uniformly distributed on the surfaces and, after ∼6 hrs, they typically aggregate into 5–8 territories. The 0 and 6 hr images presented in Fig. 4A show that the various surfaces do not alter the ability of cells to form aggregates, consistent with previous quorum-sensing experiments showing that aggregate size is actively regulated by secreted factors [38]. However, we observed that the patterns formed during the transition from individual cells to aggregates dramatically differ between the surfaces. Notably, cells plated on BSA or glass form delineated territories at 30 min that effectively coalesce into a small number of territories at 2 hrs, while cells plated on PLL or FCC do not have clearly delineated territories at 30 min and show many small aggregates at 2 hrs. In addition, cells plated on BSA form extensive streams that stretch far from aggregation centers and persist once formed. In contrast, cells on FCC or PLL immediately form small clusters of cells with minimal streaming, which come together in a coarsening process until larger aggregates are formed. Cells plated on glass form small streams (compared to cells on BSA) that eventually merge to form final aggregates. These observations were quantified by measuring the spatial extent of cell groups on each image (Fig. 4B; see Material and Methods and Fig. S4 in File S1 for more information).

These distinct behaviors could be explained if one considers that cells prefer to be in contact with each other (versus with the surface) when plated on more adhesive surfaces such as PLL or FCC. To further investigate this process, we visualized aggregating cells at high magnification with IRM and monitored the contact area with the surface. We first examined the contact areas of small clumps of ∼10 cells that form during aggregation and are observed on all surfaces. We calculated the average cell-surface contact area for cells in the group and found that it is higher when cells are plated on BSA compared to glass, which is higher than on PLL (Fig. 4C&D). Contact areas on FCC were not different from those on glass or PLL. For groups, therefore, cells tend to exhibit greater surface contact on less adhesive surfaces.

Another avenue of inquiry of collective behavior is measuring the contact area of streaming cells. Unlike for small groups, in this case the exact number of cells is unknown, and therefore the percent of the stream projected area in contact with the surface was measured. Furthermore, the incidence of streaming on PLL and FCC was very low and could not be quantified; we therefore analyzed streams formed only on BSA and glass surfaces. Here again, we found that the percentage of each stream in contact with the surface is higher when cells are plated on BSA compared to glass (Fig. 4E&F). These results suggest that during collective migration, altering the relative strengths of cell-surface and cell-cell adhesions leads to dramatically different collective patterns. The decreasing contact area with increasing cell-surface adhesion for streams and cell groups, together with the rapid clumping of cells on more adhesive surfaces, indicates that cells touching multiple surfaces tend to form greater contact area with the less adhesive surface.

## Conclusion

Together, our findings show that *Dictyostelium* cells actively control their actomyosin cortex to regulate cell-surface adhesion. Our Latrunculin A experiments show that passive cell-surface adhesivity is substantially different on the four surfaces we tested. However, cells appear to be able to actively compensate these differences with an active actomyosin cortex. Actin assembly allows cells to adhere to less adhesive surfaces, while the results from *myosin II* ^−^ cells indicates that myosin-based contractility permits cells to detach from highly adhesive surfaces. In contrast, we found that talin A is important on weak adherent surfaces, where actin is also important. Therefore, input from both actin and myosin II are required for proper adhesion regulation to allow optimal migration (see Table 1 for a qualitative summary of the results).

**Table 1:** Summary of Surface Contact Area Experiments.

	BSA	GLASS	PLL	FCC
**WT (AX3)**	= WT	= WT	= WT	= WT
**WT+LatA**	<WT	<WT	>WT	>WT
***myoII*** ^−^	= WT	= WT	>WT	>WT
***talinA*** ^−^ [*]	<WT	= WT (AX2)	>WT	= WT

“Greater Than” or “Less Than” signs indicate significantly more or less contact area than WT (p<0.05). “Equals” sign indicates no statistically significant difference from WT. The [*] for the *talinA* ^−^ cells indicates that the comparison WT cell line in that case is AX2.

Our work suggests that a mechanotransduction pathway is active during cell migration and does not require mature focal adhesions. As a result of this feedback between surface forces and the cytoskeleton, cells migrate with similar contact areas across surfaces with widely varying chemical composition. We speculate that maintaining cell-surface contact area and migration speed during chemotaxis gives *Dictyostelium* cells robustness to changing environmental conditions. Remarkably, our findings also show that, in contrast to single cell migration, collective cell migration is strongly affected by surface composition, where aggregation and streaming is dramatically inhibited on highly adhesive surfaces. These findings suggest that, in *Dictyostelium*, cell-surface contacts alter the dynamics of cell-cell interactions. We envision that a similarly complex interplay between cell-surface and cell-cell adhesion events occurs during inflammation and metastasis, where cells - either alone or in groups - use amoeboid migration to reach inflamed niches or to make it to distant sites.

## Supporting Information

File S1 (DOCX)

Movie S1 **Surface Properties Alter Self-Aggregation Dynamics.** Representative time-lapse movie of WT cells aggregating on each of the four surfaces, as filmed by dark-field microscopy. Each second represents one hour of aggregation, for a total of 6.5 hours. On BSA the aggregation process involves large streams, and on PLL and FCC aggregation involves cells clumping into small groups, which in turn coalesce into larger groups. Aggregation on glass is between these two extremes. Scale bar = 1 mm.
(AVI)
