# Supplementary material for: Dictyostelium Cells Migrate Similarly on Surfaces of Varying Chemical Composition
Source: PLoS One. 2014 Feb 6;9(2):e87981. doi: 10.1371/journal.pone.0087981 (PMC3916393; doi:10.1371/journal.pone.0087981)
Supplement: File S1 — (DOCX) [file pone.0087981.s001.docx]

**SUPPLEMENTARY DATA**

***Dictyostelium* Cells Migrate Similarly on Surfaces of Varying Chemical Composition**

**Colin P. McCann, Erin C. Rericha, Chenlu Wang, Wolfgang Losert, Carole A. Parent**

**SUPPLEMENTARY FIGURES**

**
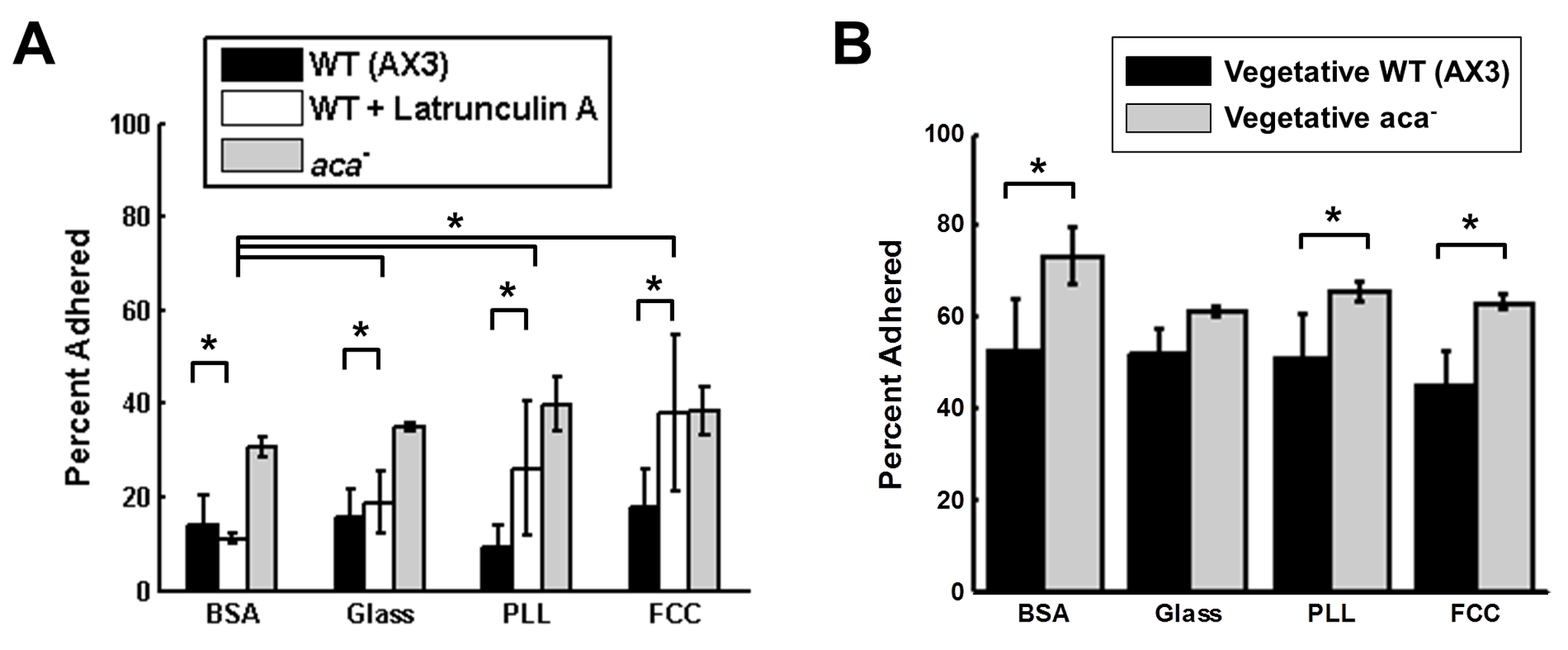
**

**Figure S1. Quantification of adhesion using the shaking adhesion assay**

**A.** Quantification of the percent adhered cells (see Material and Methods) on each surface, for WT (AX3) cells, WT cells treated with 5 μM Latrunculin A, and *aca^-^* cells. Error bars indicate SEM of three independent experiments. * indicates statistical significance (p<0.05; ANOVA, Tukey test).

**B.** Quantification of the percent adhered cells (see Material and Methods) on each surface, for vegetative WT (AX3) and *aca^-^* cells. Error bars indicate SEM of three independent experiments. Star indicates statistical significance (p<0.05; ANOVA, Tukey test).

**
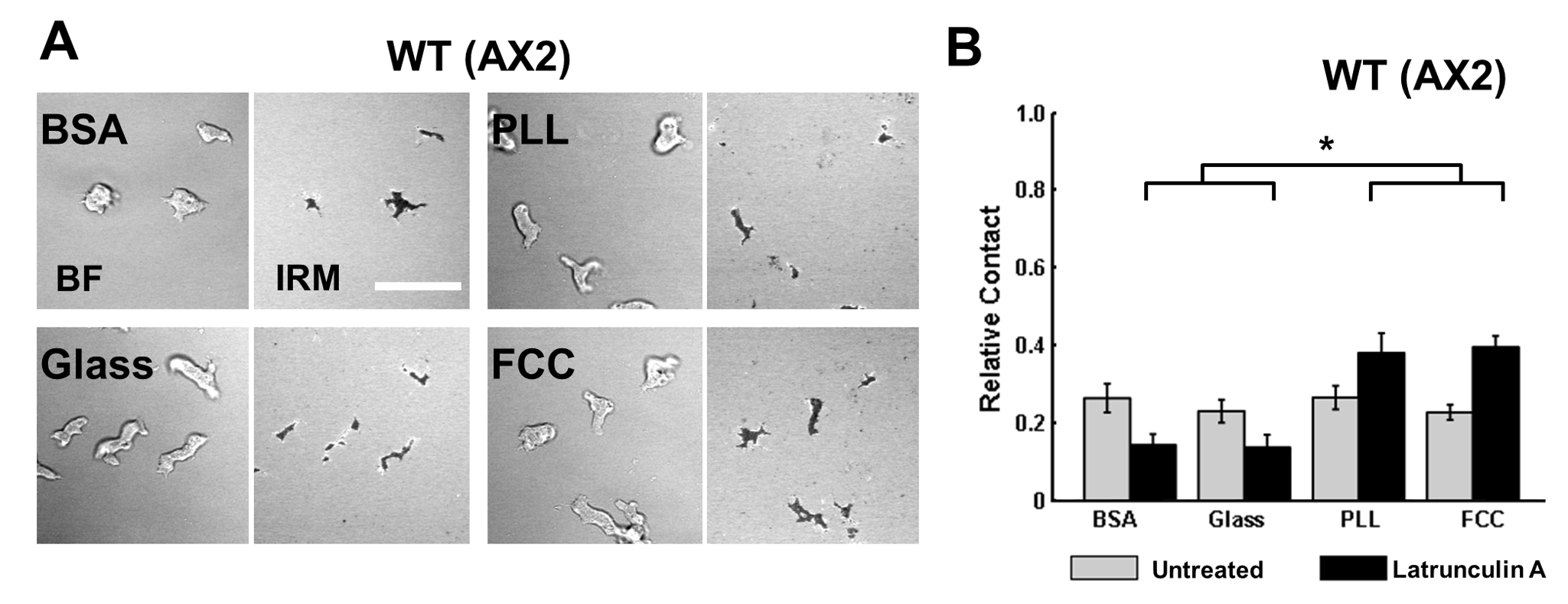
**

**Figure S2. Quantification of adhesion for AX2 cells as a control of *Talin A*^-^ cells**

**A.** Representative bright field (BF; left half of image) and IRM (right half of image) images of WT (AX2) cells on the 4 different surfaces. Scale bar = 35 μm.

**B.** Quantification of contact area of WT (AX2) cells and WT cells treated with 5 μM Latrunculin A on different surfaces. Error bars indicate SEM of three experiments. * indicate significance (p<0.05; ANOVA, Tukey test).

**
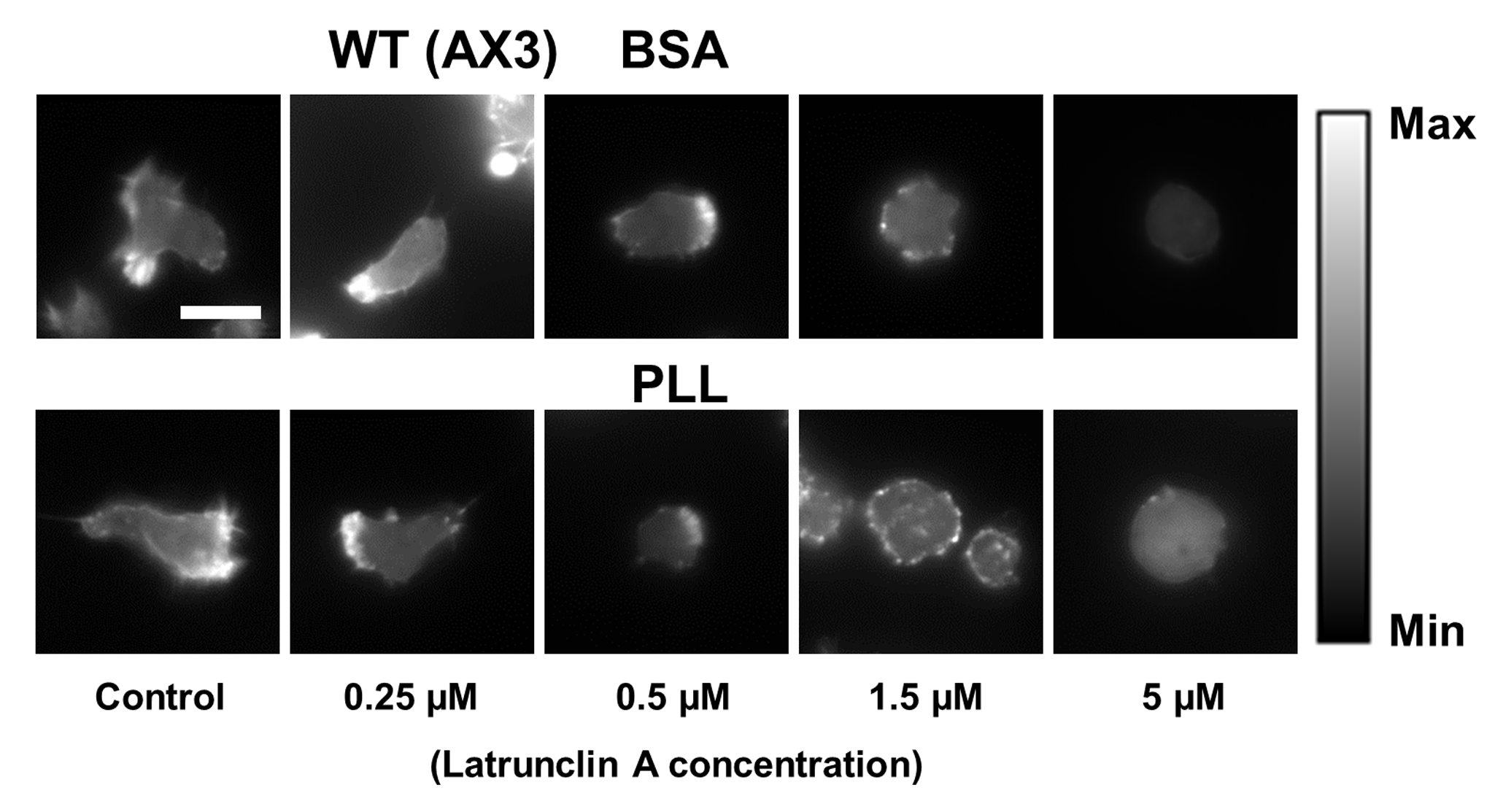
**

**Figure S3. TRITC-phalloidin staining indicate cell-surface adhesion is actin-dependent**

Representative TRITC-phalloidin fluorescent images of Latrunculin A-treated WT (AX3) cells on BSA and PLL surfaces. Scale bar = 10 μm.


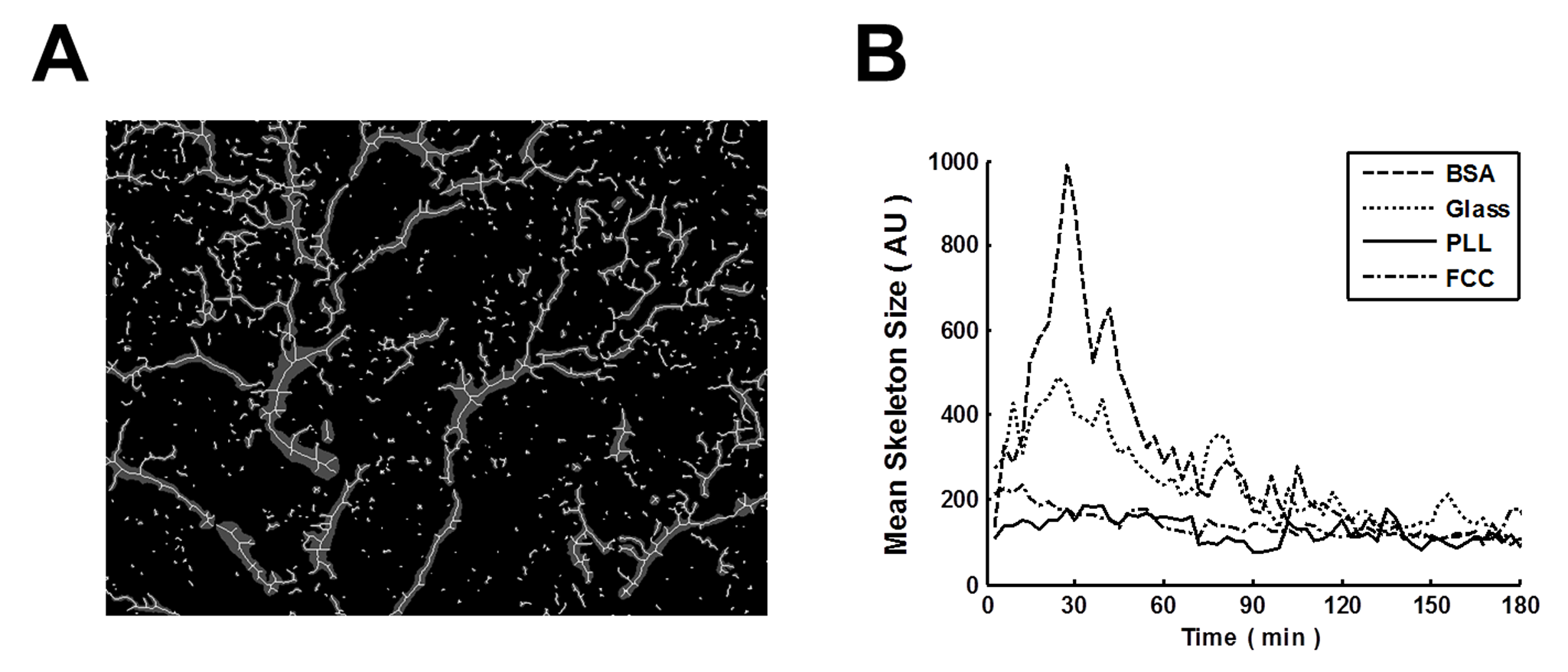


**Figure S4. Quantification of the Spatial Extent of Collective Structures**

**A.** Representative image of the quantification of object size for aggregating WT cells. The morphological skeleton (a measure of spatial extent of streams; see Material and Methods) is shown in white, with the original objects in the image shown in gray. Counting the size of the skeletons gives a relative measure of spatial object extent.

**B.** Representative quantification of spatial extent during aggregation. The morphological skeletons of the largest objects on each surface were taken as a function of time. Note that initially the absolute size of streams on BSA is larger than that on glass, which is larger than that on FCC or PLL. At the end, sizes of objects on all surfaces are similar.

**SUPPLEMENTARY TABLE**

**Table S1. Surface Properties**

| **Surface** | **Acid-Washed Glass  (Glass)** | **Bovine Serum Albumin  (BSA)** | **Poly-L-Lysine (PLL)** | **Fluorinated Carbon Chain (FCC)** |
| --- | --- | --- | --- | --- |
| **Molecular Weight** | - | 66 kDa (607 amino acids) | 30-70 kDa (144-335 amino acids) | 441 Da |
| **Charge** | Negative (Si-O^-^ exposed) | Outside is negative  (but domains have different charges) | Positive | Non-polar |
| **Hydrophobicity** | Hydrophilic | Hydrophilic | Hydrophilic | Hydrophobic |
| **Adsorption Mechanism** | - | Electrostatic and unfolds when attached to glass | Electrostatic | Covalent Si-O-Si bond |
| **Structure** | SiO_2_ | Folded amino acid polymer | Polymer of Lys | C_10_H_10_ClF_13_Si [Tridecafluoro-1,1,2,2-tetra hydrooctyl dimethyl chlorosilane] |
| **Water Contact Angle** | 10° | 40° | 35° | 120° |
